# A Liver Stiffness Measurement-Based Nomogram Predicts Variceal Rebleeding in Hepatitis B-Related Cirrhosis

**DOI:** 10.1155/2022/4107877

**Published:** 2022-06-02

**Authors:** Linxiang Liu, Qi Liu, Nanxi Xiao, Yue Zhang, Yuan Nie, Xuan Zhu

**Affiliations:** Department of Gastroenterology, The First Affiliated Hospital of Nanchang University, Jiangxi Clinical Research Center for Gastroenterology, Nanchang, Jiangxi, China

## Abstract

**Background:**

Cirrhosis esophageal variceal rebleeding is a major complication of chronic cirrhosis. The hepatic venous pressure gradient (HVPG) can predict the risk of rebleeding in patients with cirrhosis and has a good correlation with liver stiffness measurement (LSM). However, there are currently few studies based on liver stiffness to predict the risk of rebleeding in patients with liver cirrhosis. This study is aimed at exploring whether liver stiffness can predict rebleeding in patients with hepatitis B virus-related cirrhosis and developing an easy-to-use nomogram for predicting the risk of rebleeding in patients with liver cirrhosis undergoing secondary prevention.

**Methods:**

A prospective analysis of 289 cirrhosis patients was performed. Univariate and multivariate analyses were used to identify independent prognostic factors to create a nomogram. The performance of the nomogram was evaluated by using a bootstrapped-concordance index and calibration plots.

**Results:**

Use of a nonselective beta-blocker (NSBB) drug, LSM, hemoglobin, and platelet count were identified as factors that could predict rebleeding. We created a nomogram for rebleeding in cirrhosis by using these risk factors. The predictive ability of the nomogram was assessed by the *C*-index (0.772, 95% CI 0.732–0.822). The results of the calibration plots showed that the actual observation and prediction values obtained by the nomogram had good consistency.

**Conclusions:**

LSM can predict the risk of rebleeding in patients with cirrhosis, while the nomogram is a conventional tool for doctors to facilitate a personalized prognostic evaluation.

## 1. Introduction

Approximately 248 million individuals worldwide have been infected with chronic hepatitis B virus (HBV) [1], and the cirrhosis caused by it makes patients vulnerable to its complications. Bleeding from esophageal varices is a serious complication of liver cirrhosis, which brings a heavy health burden to the world. Despite the improvements in the prognosis of the disease in the past 3 decades [2], patients with cirrhosis with acute variceal bleeding (AVB) have high mortality rates of 15%-20% and a 6-week rebleeding rate of 60% in patients who have not undergone secondary prevention [3]. Therefore, there is clearly a need for simple approaches for the management of patients who will be more prone to suffer rebleeding.

The risk of bleeding from the esophagogastric veins in the setting of liver cirrhosis mainly depends on the pressure in the portal vein [4], which is manifested by the diameter of the esophageal vein and the presence of red signs. Existing studies have found that rebleeding in patients with decompensated cirrhosis is related to their baseline HVPG value [5–7]. Also, monitoring changes in HVPG values to assess response to therapy stratifies patients for their risk of rebleeding [8]. However, an invasive procedure to obtain HVPG values is not acceptable for most patients. In the diagnosis and treatment of chronic liver diseases, although HVPG provides an accurate reflection of the degree of portal hypertension, noninvasive tools are gradually introduced, mainly liver transient elastography [9].

The Baveno VI consensus recommended that the presence of varices needing treatment can be excluded in specific populations (liver stiffness measurement less than 20 kPa and platelet counts > 150000/mm^3^), because they have a low risk of first bleeding, the expanded Baveno VI standard also reached a similar conclusion [10]. Thus, liver stiffness measurement is a simple, accurate, and promising noninvasive predictor.

Liver stiffness values can predict first bleeding in patients with cirrhosis [10]. However, rebleeding from esophagogastric varices in patients with cirrhosis is often more dangerous than first bleeding. Meanwhile, previous studies have focused on an endpoint of bleeding for the first time [11, 12], and thus, there is a lack of research about rebleeding in patients with cirrhosis. Along this line, a method to predict the liver disease severity and outcomes in patients with cirrhosis is a major unmet need. In fact, after experiencing first bleeding, some patients initiate secondary prevention, and predicting the probability of rebleeding after first bleeding is critical for patient follow-up and subsequent treatment. A hypothesis has been raised that the recently proposed LSM might use to be a prognosis marker of portal hypertension. Therefore, from the perspective of reducing patients' invasive procedures, our study is aimed at determining whether liver stiffness can predict the occurrence of rebleeding events in hepatitis B virus-related decompensated cirrhosis and at producing a visual nomogram to illustrate the risk of rebleeding in cirrhosis.

## 2. Methods

### 2.1. Study Patients

This is a prospective cohort study, and consecutive hospitalized patients with liver cirrhosis were admitted to the Department of Gastroenterology, the First Affiliated Hospital of Nanchang University in China, between September 2016 and September 2020. The patient inclusion criteria were as follows: (1) age ≥ 18, (2) diagnosis of hepatitis B virus-related cirrhosis, (3) experience first bleeding and received secondary prevention of variceal rebleeding, and (4) had a liver transient elastography measurement before the second episode of variceal bleeding. The exclusion criteria included the following: (1) a diagnosis of HCC at inclusion or during the first 6 months of follow-up, (2) known HIV, (3) the first bleeding is not variceal bleeding under digestive endoscopy, (4) history of liver transplantation, (5) the patient had a large number of ascites at the time of admission or the liver function was Child C class, and (6) with severe heart and lung disease. The treatment of the included patients will be individualized according to Baveno VI standards. The study protocol was approved by the institutional ethics committee of the First Affiliated Hospital of Nanchang University (No. 2015-1206). Informed written consent was obtained from all of the study participants.

### 2.2. Clinical Data Collection and Follow-Up

Clinical data, such as age, gender, diabetes, hypertension, etiology, white blood cell, hemoglobin, platelet count, alanine aminotransferase, aspartate aminotransferase, total bilirubin, albumin, gamma-glutamyl transpeptidase, alkaline phosphatase, creatinine, international normalized ratio, prothrombin time, fibrinogen, blood urea nitrogen, HBV DNA (the lower limit of detection is 10 U/L), portal vein diameter, portal vein thrombosis, and liver stiffness measurement (LSM) were collected at admission. The Child-Pugh score and model for end-stage liver disease (MELD) score were also recorded. The data were collected independently by two physicians and checked by a third person. All included patients were followed up for rebleeding and survival. The longest follow-up period was 4 years. The primary outcome was a rebleeding event due to esophageal varices.

### 2.3. Liver Stiffness Measurement

The liver stiffness measurement was completed within 1 week after the patient underwent ligation for acute bleeding, and the patient had corrected the symptoms of hypovolemia and his vital signs were stable when they are taking liver stiffness measurements. Transient elastography was performed with FibroScan (Echosens, Paris, France) using the standard probe. LSM was considered reliable only if 10 successful acquisitions were obtained and the ratio of the interquartile range over the median (IQR/LSM) was ≤0.3. LSM was expressed in kilopascals. Patients with unreliable LSM results would have the examination repeated immediately; the results were not analyzed if they remained unreliable. The operators were blinded to all clinical data and the diagnoses of the patients.

### 2.4. NSBB Treatment and EVL Procedure

For patients receiving NSBB treatment, either carvedilol or propranolol was used. Carvedilol was started at an initial dose of 6.25 mg once daily and adjusted gradually to the maximum tolerated dose keeping heart rate > 55 beats per minute and systolic blood pressure > 90 mmHg. Propranolol was started at an initial dose of 10 mg three times daily and adjusted gradually to the maximum tolerated dose, keeping the heart rate at >55 beats per minute and the systolic blood pressure > 90 mmHg. EVL was performed using commercial multiband devices under sedation with propofol by a senior physician. The varices were ligated from the cardia to the oral side.

### 2.5. Statistical Analysis

Continuous variables are shown as the mean and standard deviation (SD) or median and interquartile range (IQR), while categorical variables are shown as frequencies (%). The rebleeding rate for the study population was generated using the Kaplan-Meier method, and differences in rebleeding rate were examined using the log-rank test. We tested whether the explanatory variable has an interaction and found no significant interactions within the included variables, such as portal vein diameter, the use of NSBB after the first bleeding, liver stiffness measurement, hemoglobin, and platelet count. Univariate and multivariate Cox models were used to calculate hazard ratios (HRs) and 95% confidence intervals (CIs) of rebleeding for patients at different time points. Forward LR selection was used to identify variables for the multivariable Cox proportional hazards regression models. All levels of significance were set at a two-sided 5% level. All analyses were performed using SPSS 25.0 IBM (IBM Corp., Armonk, NY, USA) and R 3.5.2 (R Project for Statistical Computing, Vienna, Austria). The R statistical packages “rms,” “survival,” “foreign,” and “survminer” were used to calculate the C-index and plot the calibration curves, nomogram, and Kaplan-Meier curves. Use PASS 15 (NCSS, LLC. Kaysville: Utah, USA) to calculate the sample size, with a two-sided alpha error of 0.05 and a power of 80%, and the total number of patients included in our cohort was larger than the statistically minimum sample size.

## 3. Result

### 3.1. Study Population

**Figure 1:**
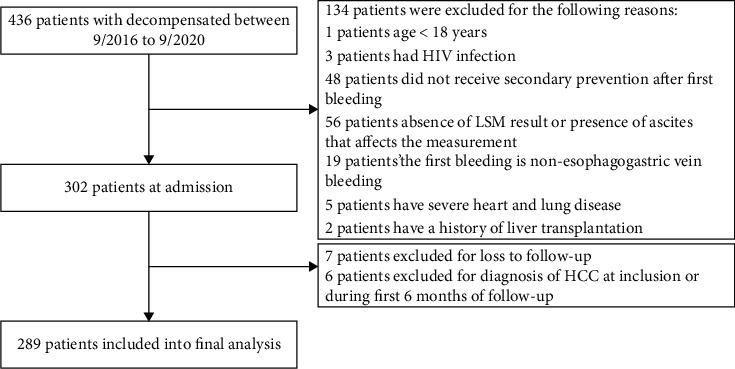
The flowchart of our study.

During the study period, a total of 289 patients with cirrhosis who received secondary prevention of variceal rebleeding and liver transient elastography measurement were included. A flowchart for the study enrollment is summarized in Figure 1. Most patients (68.86% (199 of 289)) were male. Meanwhile, Child-Pugh class “A” and “B” accounted for 91.35% and 8.65% of the patient population, respectively. In this cohort, 189 patients received either standard usage of propranolol or carvedilol combined with EVL. However, 100 patients received only EVL treatment or combined with irregular NSBB drug treatment.

During the hospitalization period when the included population had their first bleeding, 9 (3.11%) patients developed hepatic encephalopathy below stage II, 5 (1.73%) patients had fever with bacteremia or spontaneous peritonitis, and 1 (0.35%) was rebleeding within 24 hours after ligation. 122 (42.21%) patients had symptoms of hypovolemia at the time of admission, such as dizziness, increased heart rate, and decreased blood pressure. After endoscopic banding, the symptoms improved after fluid replacement/blood transfusion. These patients used vasoactive drugs such as terlipressin/somatostatin/octreotide but not epinephrine/dopamine. All patients who suffered complications during the hospitalization had fully recovered from the above complications when they are discharged.

Patients were followed up until the presence of a rebleeding episode. At a median follow-up of 66.4 weeks, rebleeding occurred in 72 out of 289 patients (24.91%). At the same time, 3 people developed hepatocellular carcinoma during the follow-up period, 4 patients underwent transjugular intrahepatic portosystemic shunt treatment (TIPS) before rebleeding, 3 people died, 2 of them from respiratory failure and 1 from hypovolemic shock during rebleeding. The clinical characteristics of the whole studied cohort are summarized in Table 1.

### 3.2. Comparison between the Rebleeding and No Rebleeding Groups

**Figure 2:**
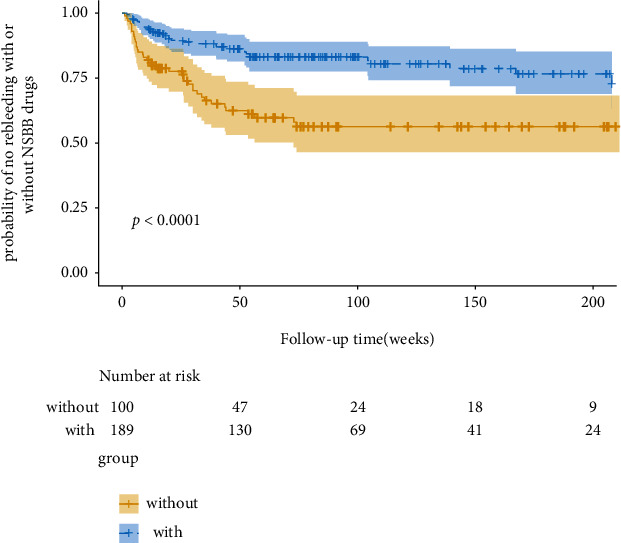
Kaplan-Meier curves demonstrating rebleeding probabilities in patients between the EVL+NSBB group and the EVL alone group. *P* values are from the log-rank test.

We compared the clinical characteristics of the two groups of patients. There were no significant differences between the rebleeding and nonrebleeding groups in terms of age, gender, BMI, portal vein thrombosis, portal vein diameter, albumin, total bilirubin, alanine aminotransferase (ALT), creatinine (Cr), blood urea nitrogen (BUN), prothrombin time (PT), MELD score, Child-Pugh score, and international normalized ratio (INR). As shown in Table 2, patients in the rebleeding group had a lower white blood cell count (4.65 ± 2.28 vs. 3.48 ± 2.08; *P* < 0.001), lower hemoglobin (103 ± 27.7 vs. 88.9 ± 24.7; *P* < 0.001), lower platelet count (104 ± 74.4 vs. 65.9 ± 38.4; *P* < 0.001), and lower fibrinogen (1.81 (1.37-2.34) vs. 1.56 (1.16-1.93); *P* = 0.012). On the other hand, liver stiffness measurement (13.3 (9.89-16.9) vs. 18.8 (13.5-23.4) *P* < 0.001) and aspartate aminotransferase (31.5 (22.8-47.5) vs. 36.0 (27.0-55.0); *P* = 0.043) were higher in the rebleeding group. Moreover, fewer patients in the rebleeding group used NSBB drugs (155 (71.4) vs. 34 (47.2); *P* < 0.001). Moreover, in order to compare the risk of recurrent bleeding in patients who only used EVL and combined with NSBB drugs, we plotted a survival probability curve. As shown in Figure 2, patients who use EVL therapy combined with NSBB drugs for secondary prevention have a significantly lower probability of rebleeding than those who use EVL alone (*P* < 0.001). Furthermore, in the EVL+NSBB group, the rebleeding rate was 17.99% (34/189), and in the EVL only group, the bleeding rate was 38% (38/100), with statistically significant difference (*P* < 0.001).

### 3.3. Prognostic Factors for the Rebleeding Rate of Patients with Cirrhosis

As shown in Table 3, we included all factors in the univariate regression analysis, and clinicopathological variables associated with the rebleeding rate were assessed a priori based on clinical importance and statistical significance. Variables include portal vein diameter, use of NSBB after the first bleeding, liver stiffness measurement, hemoglobin, platelet count, ALP, and GGT. Next, we include these 6 variables in the multivariate regression analysis and identified 4 variables, namely, use NSBB drug after the first bleeding (HR: 0.278, CI: 0.170-0.454, *P* < 0.001), liver stiffness measurement (HR: 1.026, CI: 1.005-1.048, *P* = 0.013), hemoglobin (HR: 0.986, CI: 0.977-0.995, *P* = 0.003), and platelet count (HR: 0.993, CI: 0.987-0.999, *P* = 0.018), which were independent prognostic factors for rebleeding of patients with cirrhosis.

### 3.4. Nomograms and Model Performance

**Figure 3:**
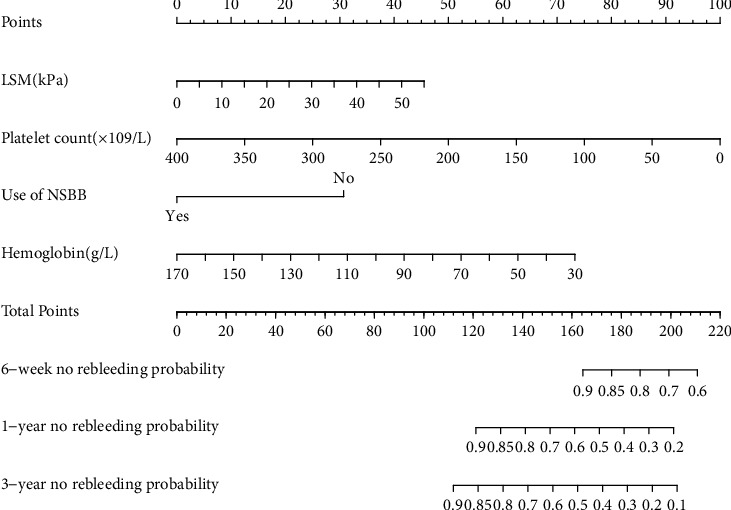
Nomogram predicting the rebleeding rate in patients with cirrhosis undergoing secondary prevention. LSM: liver stiffness measurement.

We develop a nomogram to predict rebleeding of the liver cirrhosis patients with EVL as shown in Figure 3. The nomogram to predict the rebleeding rate was created based on the following 4 independent prognostic factors: use of NSBB after the first bleeding, liver stiffness measurement, hemoglobin, and platelet count. By scoring the actual situation of each variable, higher total points based on the sum of the assigned number of points for each factor in the nomograms were associated with a worse prognosis.

**Figure 4:**
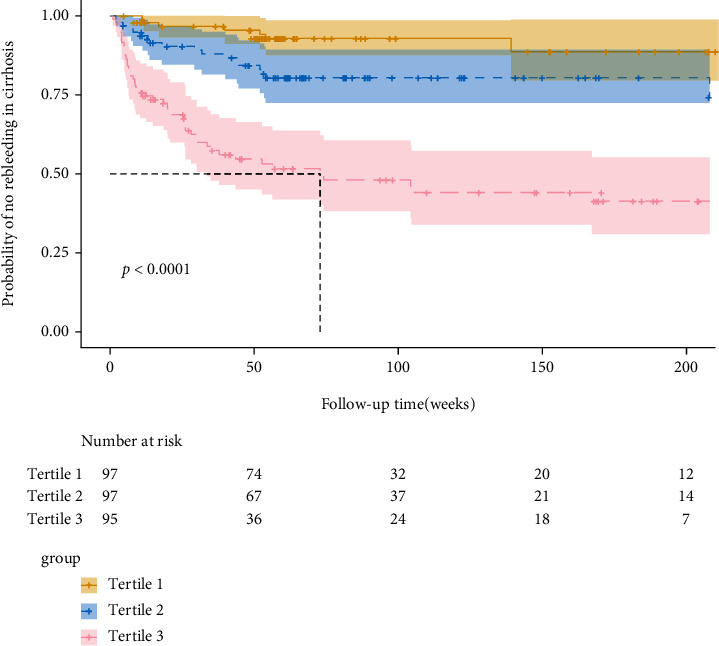
Kaplan-Meier curves demonstrating rebleeding in patients with cirrhosis undergoing secondary prevention according to tertiles of predicted rebleeding probabilities. *P* values are from the log-rank test.

**Figure 5:**
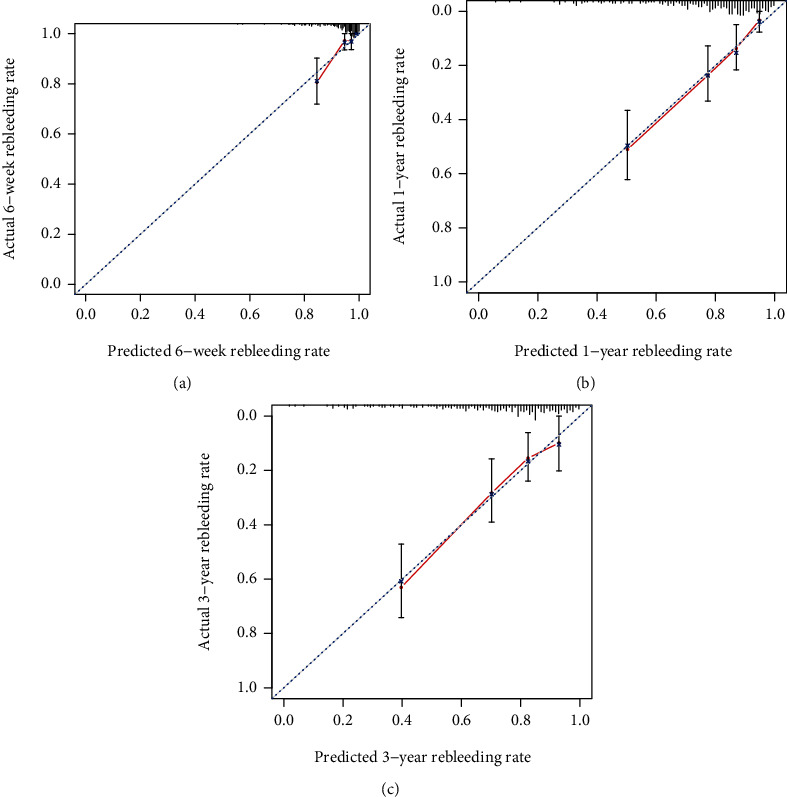
Calibration plot comparing predicted and actual rebleeding probabilities at the 6-week, 1-year, and 3-year follow-ups. (a) Calibration plot of 6-week rebleeding probabilities of the patients. (b) Calibration plot of 1-year rebleeding probabilities of the patients. (c) Calibration plot of 3-year rebleeding probabilities of the patients. The 1000-sample bootstrapped calibration plot for the prediction of 6-week, 1-year, and 3-year rebleeding probabilities is shown. The blue line represents the ideal fit; circles represent nomogram-predicted probabilities; triangles represent the bootstrap-corrected estimates; and error bars represent the 95% CIs of these estimates.

To further assess the discriminative ability of the model, the predicted probability of rebleeding of liver cirrhosis was then plotted as Kaplan-Meier curves stratified by the tertile of the predicted probability calculated from the nomograms (Figure 4). We have internally verified the prognostic nomogram. Discriminative ability in the cohort showed that the *C*-index value for the nomogram predictions of rebleeding rate was 0.772 (95% CI 0.732-0.822). These results confirm that our developed nomogram is reasonably accurate. The accuracy of the model and potential model overfit were assessed by bootstrap validation with 1000 resamplings. The 60-sample bootstrapped calibration plot for the prediction of 6-week rebleeding rate, 1-year rebleeding rate, and 3-year rebleeding rate are shown in calibration plots. The calibration plots (Figure 5) demonstrated excellent agreement between actual survival and the nomogram prediction. For example, a patient with LSM is 25 kPa, platelet count is 50 × 10^9^/L, hemoglobin is 30 g/L, used NSBB drugs would have a total of 183 points (22 points for LSM, 87 points for platelet count, 74 points for hemoglobin, and 0 point for used NSBB drugs), for a predicted 6-week no rebleeding rate, 1-year no rebleeding rate, and 3-year no rebleeding rate of 83%, 45%, and 28%, respectively.

## 4. Discussion

In this study, we present an approach that uses a nomogram based on baseline liver stiffness measurement that can predict the risk of rebleeding in patients with HBV cirrhosis who receive EVL to prevent recurrent variceal bleeding. This nomogram has good discrimination and calibration in predicting rebleeding in cirrhosis, relying only on 4 common clinical variables. We first demonstrated that the liver stiffness measurement is a clinically useful and objective tool for predicting rebleeding in patients with decompensated cirrhosis, and this gives clinicians a noninvasive way to predict the risks of these patients. At the same time, our research results may be applied to artificial intelligence platforms in the future to predict the risk of rebleeding in patients with liver cirrhosis.

It is generally recognized by researchers that the higher the patient's HVPG, the higher is the risk of bleeding they face. Monitoring the HVPG provides strong prognostic information that may be valuable for preventing rebleeding [7]. Therefore, directly reducing the HVPG pressure reduces the risk of rebleeding [13, 14]. A previous study has proposed restricting HVPG measurement to patients with ascites or hepatic encephalopathy, and measuring HVPG when the patient's baseline HVPG ≥ 16 mmHg improves the detection of high-risk patients while reducing the number of HVPG measurement required [8]. Researchers are working toward reducing invasive operations, and although some strategies in certain specific complications can reduce associated invasive operations, measuring the HVPG is an invasive procedure, which is expensive and unacceptable for most patients with cirrhosis compared with ordinary endoscopic treatment. Previous studies have reported other noninvasive methods, such as the albumin-bilirubin (ALBI) grade, platelet-albumin-bilirubin (PALBI) grade, Child-Pugh (CP) grade, and Model for End-Stage Liver Disease (MELD) score to predict the occurrence of rebleeding events, with a high *C*-index [15]. However, these four parameters only reflect the degree of liver function and do not reflect the parameters of portal vein pressure, which is the main cause of rebleeding [13].

There is a good correlation between liver stiffness measurement and portal hypertension [16–18], which makes it possible to predict liver-related events with liver stiffness measurement. Previous studies have shown that liver stiffness values can predict liver-related events in patients with cirrhosis of different etiologies [19–21]. However, liver-related events are a comprehensive concept, including hepatocellular carcinoma, portal hypertension-related decompensation, and liver-related deaths. This makes it difficult to predict the probability of a particular decompensated event of cirrhosis. Moreover, the population included in their study had compensated advanced chronic liver disease (cACLD), rather than advanced decompensated cirrhosis, and this may underestimate the probability of liver-related events in the whole liver cirrhosis population. In our study, we used a noninvasive approach to obtain the LSM. At the same time, we studied a population that had decompensated cirrhosis. They also received corresponding secondary prevention, making their situation closer to the real world. However, a subset of patients in our cohort did not receive the guideline recommendation for EVL combined with NSBB because of poor compliance. Whether in the comparison of clinical baseline variables between the bleeding group and the nonbleeding group or in the screening of risk factors in the variables, an increase in LSM had a higher chance of rebleeding. Given the correlation between LSM and HVPG, LSM, which is a promising indicator, can predict the risks of patients with liver cirrhosis and is noninvasive.

Interventions to prevent rebleeding are mandatory since mortality related to each rebleeding episode is approximately 15% to 20% [22]. The use of NSBB drugs combined with EBL is the standard prophylaxis for esophageal variceal rebleeding in cirrhosis [22, 23]. In our study, we found that patients who used NSBB had a lower rebleeding rate than those who did not. Moreover, NSBB could also reduce overall mortality [23]. Not only can NSBB reduce portal pressure and relieve hypersplenism but NSBB can also increase intestinal peristalsis and reduce the translocation of bacteria from the intestine [24]. Systemic inflammation caused by bacterial translocation can cause endothelial dysfunction and organ failure [25]. Considering that bacterial translocation is a key trigger of varicose vein bleeding, it can also increase the risk of bleeding [26].

Platelet counts have been used in scoring systems, such as the APRI, the NAFLD fibrosis score, FIB-4, King's score, GUCI, Lok index, and Forns score. When the platelet count is less than a certain threshold, the risk of rebleeding will increase [11]. Platelet dysfunction is also one of the reasons for the high incidence of rebleeding [27, 28]. However, one study found that thrombocytopenia cannot predict bleeding from esophageal varices in patients with cirrhosis [29]. Considering that the definition and exclusion criteria of liver cirrhosis in that article are not strict enough, there was a failure to exclude patients with previous platelet transfusion. At the same time, the endpoint of that study is not the occurrence of rebleeding. Therefore, researchers interpreting its results should be cautious. Platelet count may be an ideal marker for the prognosis of patients with cirrhosis when in combination with LSM. It is simple, easy to measure and process, cost-effective, and can accurately predict the severity of fibrosis.

In our study, we used baseline HB without intervention to predict the occurrence of rebleeding events at admission. Baseline HB is considered to be an independent predictor of ACLF [30]. In acute decompensation of liver cirrhosis, a low hemoglobin concentration further reduces the delivery of peripheral oxygen, which is conducive to the development of organ failure. Bacterial translocation caused by chronic inflammation, occult blood loss, and malnutrition can lead to low hemoglobin [31], thereby increasing the deterioration of liver function. Thus, the risk of rebleeding increases in cirrhosis.

Overall, our study found these parameters are related to the risk of esophageal variceal recurrent bleeding in patients with HBV cirrhosis. On the one hand, such as LSM, PLT is related to the patient's portal hypertension. There is no doubt that patients with high portal pressure are more prone to bleeding again. On the other hand, hemoglobin, use of NSBB is considered to be related to systemic hemodynamics. Low hemoglobin causes systemic peripheral vasoconstriction, liver hypoxia, and heart damage, all of which can aggravate hemodynamic disorders [32]. Using NSBB to obtain a hemodynamic response can significantly reduce the occurrence of rebleeding events. Hence, the above variables fully explain their prognostic value in patients with liver cirrhosis and rebleeding.

Our nomogram is a simple visual tool that can be used to predict the occurrence of rebleeding in patients with liver cirrhosis. It also has excellent discrimination and calibration. To our knowledge, this is the first nomogram to predict rebleeding in patients with decompensated cirrhosis based on baseline liver stiffness. Before treatment, a nomogram can be used to predict the probability of rebleeding. After treatment, the nomogram can help doctors distinguish high-risk and low-risk patients, and high-risk patients should be followed up carefully.

The main limitation of this study lies in the nomogram which was estimated using prospective data from a single center and lack of an external validation cohort. This result needs to be confirmed in a larger, ethnically, and geographically more diverse population. Another relevant limitation is, in patients using NSBB drugs, we failed to detect their hemodynamic changes and could not accurately explain its relationship with the occurrence of rebleeding. However, our research still found that patients using NSBB can reduce their risk of rebleeding. Similarly, our study failed to record the change in LSM during follow-up. Although the current study suggests that the change in LSM has an impact on patient prognosis. However, our study focused on the significance of baseline LSM for the prognosis of liver cirrhosis and obtained reliable results. Finally, the patients in this study are HBV-related patients with Child-Pugh A/B and without large size of ascites, and this might limit the use of this nomogram in clinical practice.

In conclusion, we developed and validated a nomogram for predicting the recurrent hemorrhage in cirrhosis. This simple nomogram had an adequate ability of discrimination and calibration. It could be a useful tool for patients undergoing a preoperative consultation and for doctors conducting a postoperative evaluation. Moreover, this may be a promising noninvasive assessment tool for predicting rebleeding in patients with liver cirrhosis, and it can be used in clinical practice to avoid unnecessary HVPG and endoscopic operations.

## Figures and Tables

**Table 1:** Clinical characteristics of study participants. Patient characteristics.

	All patients (*n* = 289)
Gender	
Male	199 (68.86)
Female	90 (31.14)
Age (years)	55.96 (13.15)
BMI (kg/m^2^)	22 (3.07)
Hypertension	27 (9.34)
Diabetes	36 (12.46)
HB (g/L)	99.5 (27.6)
WBC (×10^9^/L)	4.36 (2.28)
Use of NSBB	189 (65.4)
PTV	36 (12.46)
AST (IU/L)	35 (26-52.5)
ALT (IU/L)	24 (16-40.5)
Platelet count (×10^9^/L)	71 (46-121.5)
Total bilirubin (*μ*mol/L)	19.5 (12.8-29.6)
Albumin (g/L)	34.7 (6.08)
ALP (IU/L)	88 (62-130)
GGT (IU/L)	32 (19-74)
Baseline LSM (kPa)	14.25 (10.58-19.13)
PT (second)	13.6 (12.6-14.8)
INR	1.21 (1.11-1.33)
Fibrinogen (g/L)	1.97 (1.15)
Cr (*μ*mol/L)	65.5 (55.8-77.9)
BUN (mmol/L)	5.9 (5.19)
HBeAg	59 (20.41)
HBV DNA (log_10_ IU/mL)	4.67 ± 1.68
Portal vein diameter (cm)	1.5 (1.3-1.6)
MELD score	9.04 (8.126-11.17)
Child-Pugh class	
A	264 (91.35)
B	25 (8.65)

Continuous variables are expressed as mean (standard deviation) or median (interquartile range); categorical variables are expressed as number (percentage). BMI: body mass index; HB: hemoglobin; WBC: white blood cell; PVT: portal vein thrombosis; ALT: alanine aminotransferase; AST: aspartate aminotransferase; ALP: alkaline phosphatase; GGT: gamma-glutamyl transpeptidase; LSM: liver stiffness measurement; INR: international normalized ratio; Cr: creatinine BUN: blood urea nitrogen; PT: prothrombin time.

**Table 2:** Clinical characteristics between the rebleeding and nonrebleeding groups.

	No rebleeding (*n* = 217)	Rebleeding (*n* = 72)	*P* value
Age (years)	49.82 (12.99)	53.17 (12.09)	0.055
Male	149 (68.66)	53 (73.61)	0.379
BMI	21.86 (3.04)	22.4 (3.13)	0.193
Portal vein diameter (cm)	1.4 (1.3-1.6)	1.5 (1.3-1.6)	0.100
Use of NSBB	155 (71.4)	34 (47.2)	**<0.001**
Baseline LSM (kPa)	13.3 (9.89-16.9)	18.8 (13.5-23.4)	**<0.001**
WBC (×10^9^/L)	4.65 (2.28)	3.48 (2.08)	**<0.001**
HB (g/L)	103 (27.7)	88.9 (24.7)	**<0.001**
Platelet count (×10^9^/L)	104 (74.4)	65.9 (38.4)	**<0.001**
PT (second)	13.4 (12.5-14.8)	14.0 (12.7-14.8)	0.235
INR	1.19 (1.10-1.33)	1.25 (1.14-1.34)	0.058
Fibrinogen (g/L)	1.81 (1.37-2.34)	1.56 (1.16-1.93)	**0.012**
Albumin (g/L)	35.0 (6.29)	35.1 (5.42)	0.826
Total bilirubin	18.9 (12.3-31.9)	20.0 (13.7-28.8)	0.974
ALT (U/L)	21.0 (15.8-31.8)	25.0 (17.0-45.0)	0.07
AST (U/L)	31.5 (22.8-47.5)	36.0 (27.0-55.0)	**0.043**
Cr (*μ*mol/L)	5.98 (5.25)	5.65 (5.02)	0.634
BUN (mmol/L)	65.0 (56.0-77.2)	6.2 (55.3-80.6)	0.887
MELD score	9.07 (8.13-11.4)	9.54 (8.31-11.3)	0.151
Child-Pugh class			0.229
A	205 (70.9%)	56 (19.7%)	
B	12 (4.2%)	16 (5.2%)	

*P* value < 0.05 indicates a significant difference between the cohorts presenting with rebleeding event versus no rebleeding. BMI: body mass index; HB: hemoglobin; WBC: white blood cell; PVT: portal vein thrombosis; ALT: alanine aminotransferase; AST: aspartate aminotransferase; ALP: alkaline phosphatase; GGT: gamma-glutamyl transpeptidase; LSM: liver stiffness measurement; INR: international normalized ratio; Cr: creatinine BUN: blood urea nitrogen; PT: prothrombin time.

**Table 3:** Prognostic factors for rebleeding rate of cirrhosis patients.

	Univariate		Multivariate	
	HR (95% CI)	*P* value	HR (95% CI)	*P* value
Gender	1.267 (0.75-2.14)	0.376		
Age (years)	0.987 (0.97-1.003)	0.113		
BMI	1.054 (0.981-1.132)	0.153		
PVT	0.662 (0.355-1.234)	0.194		
Portal vein diameter (cm)	2.458 (1.159-5.211)	0.019		
Use of NSBB	0.387 (3.392-0.615)	0.000	0.278 (0.17-0.454)	**0.000**
Baseline LSM (kPa)	1.03 (1.01-1.049)	0.003	1.026 (1.005-1.048)	**0.013**
WBC (×10^9^/L)	0.936 (0.853-1.028)	0.169		
HB (g/L)	0.983 (0.974-0.992)	0.000	0.986 (0.977-0.995)	**0.003**
Platelet count (×10^9^/L)	0.988 (0.983-0.994)	0.000	0.993 (0.987-0.999)	**0.018**
PT (second)	1.015 (0.967-1.066)	0.543		
INR	1.006 (0.74-1.368)	0.970		
Fibrinogen (g/L)	0.92 (0.714-1.187)	0.522		
Albumin (g/L)	1.005 (0.968-1.043)	0.791		
Total bilirubin (*μ*mol/L)	1 (0.988-1.011)	0.934		
ALT (IU/L)	0.997 (0.991-1.003)	0.379		
AST (IU/L)	0.998 (0.993-1.003)	0.372		
GGT (IU/L)	0.997 (0.995-1)	0.062		
ALP (IU/L)	0.997 (0.994-1)	0.08		
BUN (mmol/L)	0.987 (0.935-1.042)	0.653		
Cr (*μ*mol/L)	0.996 (0.987-1.006)	0.456		
MELD score	1.048 (0.981-1.12)	0.161		
Child-Pugh class				
A		Ref		
B	0.692 (0.399-1.2)	0.190		

All variables were entered in a forward LR elimination procedure with a *P* value to exit set at >0.10. Empty cells refer to the variables excluded from the multivariable-adjusted logistic regression models. BMI: body mass index; HB: hemoglobin; WBC: white blood cell; PVT: portal vein thrombosis; ALT: alanine aminotransferase; AST: aspartate aminotransferase; ALP: alkaline phosphatase; GGT: gamma-glutamyl transpeptidase; LSM: liver stiffness measurement; INR: international normalized ratio; Cr: creatinine BUN: blood urea nitrogen; PT: prothrombin time.

## Data Availability

The data used to support the findings of this study are available from the corresponding author upon request.
